# Hyaluronate gel injection for rectum dose reduction in gynecologic high-dose-rate brachytherapy: initial Japanese experience

**DOI:** 10.1093/jrr/rrz016

**Published:** 2019-04-29

**Authors:** Tairo Kashihara, Naoya Murakami, Nikolaos Tselis, Kazuma Kobayashi, Keisuke Tsuchida, Satoshi Shima, Koji Masui, Ken Yoshida, Kana Takahashi, Koji Inaba, Rei Umezawa, Hiroshi Igaki, Yoshinori Ito, Tomoyasu Kato, Takashi Uno, Jun Itami

**Affiliations:** 1 Department of Radiation Oncology, National Cancer Center Hospital, Tsukiji 5-1-1, Chuo-ku, Tokyo, Japan; 2 Department of Radiotherapy and Oncology, University of Frankfurt, Theodor-W.-Adorno-Platz 1 , Frankfurt am Main, Germany; 3 Department of Radiology, Kyoto Prefectural University of Medicine, Kawaramachi-Hirokoji, Kamigyo-ku, Kyoto, Japan; 4 Department of Radiation Oncology, Osaka Medical College, Daigakucho 2-7, Takatsuki, Osaka, Japan; 5 Department of Gynecologic Oncology, National Cancer Center Hospital, Tsukiji 5-1-1, Chuo-ku, Tokyo, Japan; 6 Department of Radiology, Chiba University Hospital, 1-8-1 Inohana, Chuo-ku, Chiba-shi, Chiba, Japan

**Keywords:** gynecologic malignancies, brachytherapy, high-dose rate, hyaluronate gel

## Abstract

Perirectal hyaluronate gel injection (HGI) appears to be a promising technique for healthy tissue dose sparing in pelvic radiotherapy. In this analysis, we report our initial experience of HGI in gynecologic brachytherapy, focusing on its safety and effectiveness for dose reduction to the rectum. Between July 2013 and May 2014, 36 patients received HGI for primary/salvage gynecologic brachytherapy. Dosimetric effect analysis was based on pre- and post-HGI computed tomography dataset registration with corresponding dose–volume histogram evaluation. The maximum dose to the most exposed 0.1 cm^3^ (D_0.1__cm^3^_) and 2.0 cm^3^ (D_2.0__cm^3^_) were used as index values for rectum and bladder dose evaluation. The dose indexes for target volume (TV) coverage were TV D90/V100. In all cases, HGI was well tolerated, with no acute or late adverse events documented at a median follow-up of 220 days (range, 18–1046 days). Rectum D_2.0__cm^3^_ and D_0.1__cm^3^_ were significantly decreased by HGI (*P* < 0.001 and *P* = 0.003, respectively), with no significant impact on dosimetric parameters of bladder and TV coverage. Factors correlating negatively with the dosimetric effect of HGI were an increasing number of interstitial catheters (*P* = 0.003) as well as Lcranial100% (*P* = 0.014) and Lcranial80% (*P* = 0.001) [i.e. the length from the anal verge to the most cranial point at which the 100% and 80% isodose lines, respectively, crossed the rectum]. The concept of HGI for gynecologic brachytherapy is plausible, and our initial experience indicates it to be an effective technique for rectal dose reduction in radiotherapy of intrapelvic tumours.

## INTRODUCTION

Despite significant improvements in the management of gynecologic malignancies, the major pattern in failure following radical therapy remains locoregional [1, 2], with the factors affecting retreatment including the extent of recurrent disease, the primary surgical technique, and previously administered radiotherapy (RT). In addition, adverse effects on the rectum, such as rectovaginal fistula formation, are major problems in gynecological brachytherapy [3]. In particular, radical percutaneous reirradiation is usually not feasible because of excessive morbidity on account of previous external beam radiotherapy (EBRT). On the other hand, recent clinical research suggests that radiation dose escalation in the primary treatment of localized gynecologic tumors results in improved local control with brachytherapy (BT), enhancing the therapeutic ratio by escalating the treatment dose while ameliorating dose conformity [4, 5]. In particular, high-dose-rate (HDR) BT meets this objective optimally by exploiting the radiobiological advantage of larger fraction sizes while ensuring prospective 3D dosimetry [6]. As such, 3D HDR BT for cervical cancer has shown dosimetric superiority, allowing for a paradigm shift from traditional point A dosimetry to anatomy-oriented treatment planning [7, 8]. Against this background, HDR BT appears to be a promising RT modality, particularly with regard to the treatment of recurrent gynecologic malignancies [9–11], due to its potential to reduce the dose to organs at risk (OARs) [12].

Notwithstanding this, image-based dose optimization might still pose a clinically relevant challenge when the anatomy of OARs hampers the application of sufficient doses, either in the primary or in the recurrent treatment setting [13]. In this regard, perirectal hyaluronate gel injection (HGI) has been shown to be an effective technique for rectal dose reduction in EBRT and BT of localized prostate cancer [14–23], with some experiences also documenting its use in the treatment of bulky vaginal stump recurrences of uterine cancer [24]. Two small-sized case series have been reported so far on the use of HGI for intracavitary application (ICBT) in gynecologic BT, but one was a study of three patients with locally recurrent gynecologic malignancies requiring reirradiation [25], and the other was a study of only five cadavers who did not show cancer in the uterine cervix [26]. In this retrospective analysis, we report our initial experience with HGI in ICBT and interstitial BT (ISBT) of living gynecological cancer patients, focusing on its safety and effectiveness for dose reduction to OARs.

## MATERIALS AND METHODS

### Patients

Between July 2013 and May 2014, 36 consecutive patients who received HGI for subsequent gynecologic BT were identified. Twenty-eight patients presented with cervical cancer, six patients with uterine cancer, 1 patient with vaginal cancer, and 1 with vulvar melanoma. BT was applied as the primary treatment in 16 cases, and 20 patients received it as salvage treatment. Among these, previous surgery was performed in 10 patients, and RT with or without surgery in the remaining 10. Treatment prior to BT included EBRT with a median dose of 73.2 Gy (range, 50.0–81.2 Gy) in 10 patients. In all cases, HGI was deemed to be indicated due to anatomical conditions resulting in excessive radiation dose exposure to the relevant OARs (i.e. the rectum, bladder and vagina). These included patients with a narrow vaginal canal that did not allow adequate packing to spare the rectum dose, patients receiving reirradiation for recurrent disease in close proximity to OARs, as well as patients with very high-dose vaginal mucosa exposure in cases involving dose escalation without spacer injection. BT was the sole treatment in 22 patients, whereas 14 patients also received whole-pelvic RT (WPRT) combined with BT. The median EBRT dose was 50.0 Gy (range, 48.4–50.0 Gy). The treatment was performed as ISBT in 23 patients and ICBT in the remaining 13 cases. Patient characteristics are summarized in Table 1. Informed consent was obtained from the patients prior to the treatment, which was performed with standard institutional approval.

**Table 1.** Patient characteristics

Characteristic	*N*
*Age (years), median (range)*	63 (range, 39–85)
*Follow-up time (days), median (range)*	220 (range, 18–1046)
*Primary tumour site*	
Cervix	28
Uterine body	6
Vagina	1
Vulvae	1
*Treatment indication*	
Primary treatment	16
Cervix clinical T stage 1B1	1
2A1	1
2B	1
3 A	1
3B	1
4 A	2
4B	2
Vagina clinical T stage 2	1
Salvage treatment	20
Previous surgery	10
Cervix	6
Uterine body	3
Vulvae	1
Previous RT	5
Cervix	5
Previous surgery and RT	5
Cervix	2
Uterine body	3
*Pathology*	
Squamous cell carcinoma	22
Adenocarcinoma	11
Adenosquamous	2
Melanoma	1
*Type of brachytherapy*	
Intracavitary brachytherapy	13
Interstitial brachytherapy	23
*Number of applicators, mean (range)*	16 (1–28)

RT = radiotherapy.

### Method of HGI

**Fig. 1.**
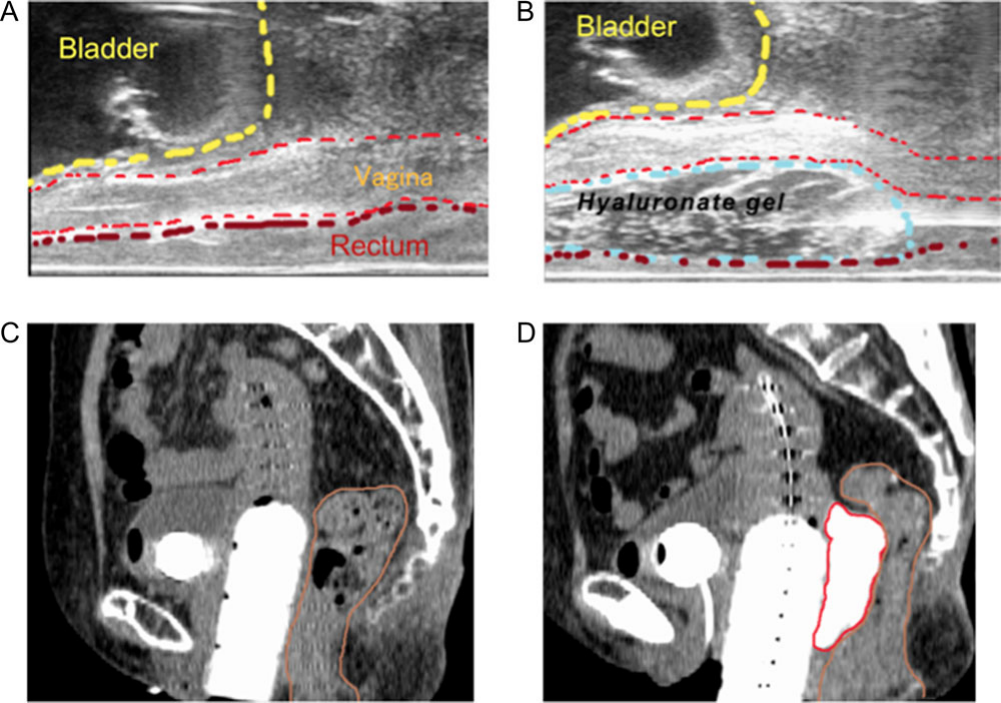
Hyaluronate gel injection (HGI) is performed under real-time transrectal ultrasound (TRUS) guidance. (A) Sagittal TRUS image obtained before HGI. (B) Sagittal TRUS image obtained during HGI. An application needle is inserted between the vagina and rectum via a transperineal approach, and hyaluronate gel is being injected into the pararectal space. Sagittal CT images before HGI (C) and after HGI (D) are also shown. The red line marks the hyaluronate gel and the brown line the rectum.

Our technique of HGI has been described by Kishi *et al.* [14, 24]. In short, HGI was performed under local anesthesia and intravenous sedoanalgesia, using a transperineal approach with real-time transrectal ultrasound (TRUS) guidance. The puncture point was the lateral side of the labium major or anal ring. The needles were inserted through subcutaneous adipose tissue, perineal fascia and muscle (superficial transverse perineal muscle and transverse vaginal muscle), and the levator ani muscle to reach the desired point of the pararectal space. For identification of the injection site, the muscularis propria of the rectum, which was visible as a hypoechoic dark band on axial and sagittal imaging, was approached. Once the needle tip was confirmed to be between the vagina and rectum using both axial and sagittal images, 1–3 cm^3^ of hyaluronate gel was injected gently. After separation between the vagina and rectum had been confirmed, additional hyaluronate gel was injected. Figure 1 shows the TRUS-based injection procedure. We injected 5–20 cm^3^ of hyaluronate gel (~1 mg/cm^3^, Suvenyl, Chugai/Roche, Tokyo, Japan) diluted with 5–20 cm^3^ of saline solution and 1–2 cm^3^ of contrast dye (Iopamiron, Bayer, Leverkusen, Germany), and up to a total volume of 10–40 cm^3^ was injected. The median amount of gel injected was 20 cm^3^. In each case, the decision regarding the amount and interventional approach was based on the experience of the treating physician.

### Analysis of dosimetric effects of HGI

For analysis of the dosimetric impact of HGI, pre- and post-HGI computed tomography (CT) scans were used in order to calculate the volume and the radiation exposure of the rectum and the bladder before and after HGI. The first CT scan was taken after implantation of interstitial/intracavitary catheters and before HGI. The second CT scan was taken after completion of HGI, with the catheters *in situ*. The rectum was defined as extending from the anal verge to the sigmoid flexure, and the whole volume of the bladder was contoured in the planning CT. Coverage of the target volume (TV) was also calculated and analyzed. In cases involving primary treatment for cervical cancer BT, the high-risk clinical target volume (HR-CTV) [12] was used as the TV. Pre- and post-HGI CT images were registered using the point-based registration technique [27]. The maximum dose to the most exposed 0.1 cm^3^ (D_0.1__cm^3^_) and the most exposed 2.0 cm^3^ (D_2.0__cm^3^_) of the organ were used as index values for the rectum dose evaluation [28, 29]. The dose indexes for the bladder dose as well as the TV coverage were Bladder D_2.0__cm^3^_, Bladder D_0.1__cm^3^_, TV D_90_ and TV V_100_ [V_100_ = percentage of the clinical target volume (CTV) receiving 100% of the prescription dose, D_90_ = minimum dose covering 90% of the CTV]. In addition, factors affecting the rate of variability of the Rectum D_2.0__cm^3^_ were also investigated. For this purpose, the rate of variability of the Rectum D_2.0__cm^3^_ was calculated using the following equation:

$$\begin{array}{c} {\mathrm{Rate of Rectum D}}_{2\mathrm{.0}{\mathrm{cm}}^{3}}\mathrm{variability}=\\ ({\mathrm{pre}\text{-}\mathrm{HGI Rectum D}}_{2\mathrm{.0}{\mathrm{cm}}^{3}}{-\mathrm{post}\text{-}\mathrm{HGI Rectum D}}_{2\mathrm{.0}{\mathrm{cm}}^{3}})/\\ {\mathrm{pre}\text{-HGI Rectum D}}_{2\mathrm{.0}{\mathrm{cm}}^{3}} \end{array}$$

**Fig. 2.**
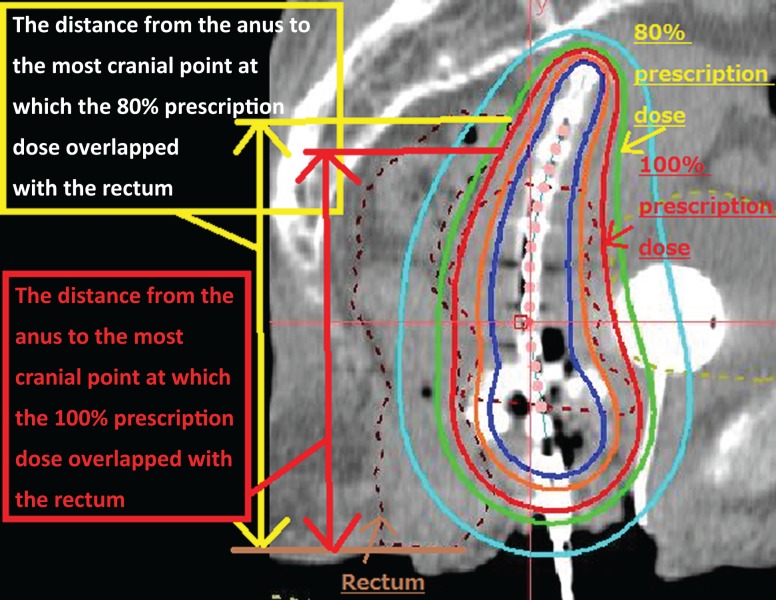
Pre-HGI CT image in sagittal view. The length from the anal verge to the most cranial point at which the 100% and 80% isodose line crossed the rectum on the pre-HGI image were defined as L_cranial100%_ and L_cranial80%_, respectively. The red line indicates the 100% isodose line and the green line the 80% isodose line. The brown dotted line marks the rectum.

For evaluation of the factors modifying the Rectum D_2.0__cm^3^_ variability, the lengths from the anal verge to the most cranial point at which the 100% and 80% prescription isodose lines crossed the rectum (L_cranial 100%_ and L_cranial 80%_, respectively) were measured on sagittal CT images (Fig. 2). For patients who received BT more than once, the mean value of the parameters was calculated. In addition, we analyzed whether the rectum dose constraint of Rectum D_2.0__cm^3^_ < 70 Gy [30] was satisfied before and after HGI in patients without a prior history of irradiation.

### Brachytherapy

Our procedure for ISBT in patients with cervical cancer has been described in detail elsewhere [31–33]. In short, interstitial catheter insertion was performed transperineally under TRUS-guidance, and simulation CT was carried out using a large-bore CT unit (Aquilion LG, Toshiba, Tokyo, Japan), with patients in the lithotomy position and catheters *in situ.* Treatment planning was performed with dedicated BT planning software (Oncentra, Nucletron, Veenendaal, the Netherlands), and all treatments were performed using a ^192^Iridium remote afterloading system (RALS, Microselectron HDRTM, Nucletron, Veennendaal, The Netherlands). Intracavitary BT was performed after implementation of tandem and ovoid applicators, using the same planning procedure. The prescribed reference dose per fraction (100% isodose) was 6 Gy in all cases. In cases involving multifraction implants, CT-based planning was performed once for all treatment fractions. In every other setting, treatment planning was performed for each treatment.

### Follow-up and statistical analyses

After treatment, all patients were followed-up every 1–3 months in order to observe and document possible adverse events using the Common Terminology Criteria for Adverse Events version 4.0. Comparisons between pre- and post-HGI doses of the rectum and bladder as well as the TV coverage were performed using the paired *t*-test [34]. In addition, Tukey’s honestly significant difference test [35] was performed to evaluate the association between the rate of variability of the Rectum D_2.0__cm^3^_ and selected clinical factors. All statistical tests were two-sided, and the level of statistical significance was defined as *P* < 0.05. Statistical analyses were performed using IBM SPSS Statistics (v. 19.0.0) (IBM Corp., Armonk, NY, USA) and SAS (version 9.2, Institute Inc., Cary, NC, USA).

### Ethical approval and permission for off-label use of hyaluronate gel

All procedures involving human participants performed in this study were approved by the institutional research committee (Approval number: 2017-091) and were in accordance with the ethical standards of the committee and with the 1964 Declaration of Helsinki and its later amendments or comparable ethical standards.

In addition, an internal review of this application was performed by a committee formed under the Pharmaceutical Affairs Committee, which was under the jurisdiction of the Hospital Director. The committee for internal review was independent of the research ethics review committee and consisted of five members, including physicians, pharmacists, and staff of the medical affairs division.

## RESULTS

In all cases, HGI was well tolerated and associated with only minor discomfort not necessitating any type of treatment or intervention. No acute or late adverse events more than Grade 3 related to the spacer injection were documented. The median follow-up period was 711 days (range, 71–1326 days). On the other hand, some patients experienced severe toxicities as a result of irradiation. No acute or late gastrointestinal (GI) or genitourinary (GU) toxicities were detected in patients undergoing upfront BT, but two patients who received salvage BT after prior RT experienced severe adverse events. One patient experienced Grade 3 rectal bleeding and the other showed rectovaginal and vesicovaginal fistulas.

In order to compare the dose to the rectum and bladder as well as the TV coverage before and after HGI, pre**-** and post**-**HGI CT scans along with the corresponding dose–volume histograms were analyzed. CT scans were available in all but eight patients, who were therefore excluded from this analysis. The mean error value for image registration pre- and post-HGI CT scans was 1.84 mm (range: 0.8–2.9 mm). As shown in Table 2, the Rectum D_2.0__cm^3^_ and D_0.1__cm^3^_ were significantly decreased by HGI (*P* < 0.001 and *P* = 0.003, respectively). The mean dose difference for the Rectum D_2.0__cm^3^_ and D_0.1__cm^3^_ between pre-HGI and post-HGI was 110.5 ± 14.9 cGy and 111.0 ± 38.0 cGy (mean ± SE), respectively. On the other hand, the evaluated dosimetric parameters of bladder and TV coverage were not significantly different. There was also no significant change related to the volume of the rectum and bladder (Table 3).

**Table 2.** Dose parameters affected by HGI

Parameters	Dosimetric value (median, range)		Effect of HGI
	Pre-HGI	Post-HGI	*P* value
Rectum D_2.0__cm^3^_	484.67 cGy	369.63 cGy	*P* < 0.001
	(322.76–665.26 cGy)	(255.81–575.72 cGy)	
Rectum D_0.1__cm^3^_	613.89 cGy	491.49 cGy	*P* = 0.003
	(422.45–827.58 cGy)	(335.32–758.89 cGy)	
Bladder D_2.0__cm^3^_	506.71 cGy	488.62 cGy	*P* = 0.278
	(209.39–733.92 cGy)	(209.39–779.79 cGy)	
Bladder D_0.1__cm^3^_	665.23 cGy	607.50 cGy	*P* = 0.270
	(323.67–1202.85 cGy)	(323.67–869.70 cGy)	
HR-CTV D_90_	658.82 cGy	660.37 cGy	*P* = 0.953
	(574.29–743.16 cGy)	(568.66–777.55 cGy)	
HR-CTV V_100_	95.36%	95.45%	*P* = 0.173
	(94.97–99.99 %)	(95.39–99.99 %)	

D_0.1__cm^3^_ = maximum dose to the most exposed 0.1 cm^3^ of the organ, D_2.0__cm^3^_ = maximum dose to the most exposed 2.0 cm^3^ of the organ, HR-CTV D_90_ = minimum dose covering 90% of the high-risk CTV, HR-CTV V_100_ = percentage of the high-risk CTV receiving 100% of the prescription dose.

**Table 3.** Volume change of the rectum and bladder hyaluronate gel injection

	Pre-HGI	Post-HGI	*P* value
Rectum volume	79.08 cm^3^	63.03 cm^3^	*P* = 0.297
Median (range)	(37.40–154.18 cm^3^)	(27.88–105.97 cm^3^)	
Bladder volume	191.41 cm^3^	205.41 cm^3^	*P* = 0.423
Median (range)	(35.30–696.46 cm^3^)	(35.30–389.03 cm^3^)	

HGI = hyaluronate gel injection. Rectum was defined from anal verge to sigmoid flexure. Whole bladder volume was defined as bladder.

**Fig. 3.**
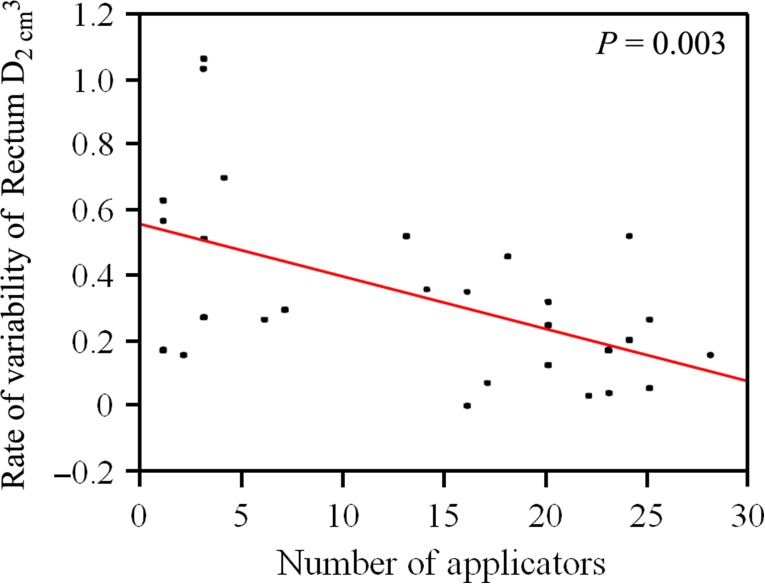
Correlation diagram of Rectum D_2.0__cm^3^_ and the number of implanted catheters. It shows that with an increase in the number of catheters used, the dosimetric effect of HGI reduces.

**Fig. 4.**
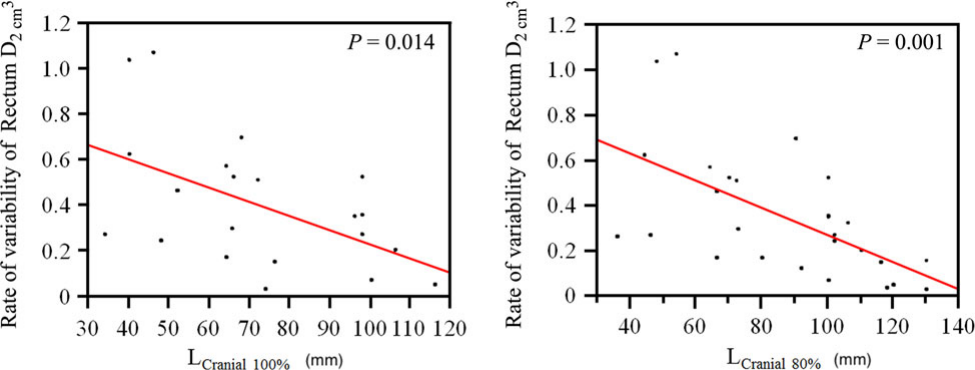
Correlation diagram of Rectum D_2.0__cm^3^_ and L_Cranial100%_ and L_cranial80%_. It shows that the more cranial the high-value isodose lines overlapping the rectum, the less is the dosimetric effect of HGI.

Of note, in 14 out of the 15 patients with cervical cancer receiving BT as the primary treatment, the total number of BT sessions and the applied total physical treatment dose could be increased after HGI, compared with the recommendations by the Japanese guidelines for the treatment of cervical cancer [36]. In patients without a prior history of irradiation, 13 patients (50.0%) did not satisfy the dose constraint of Rectum D_2.0__cm^3^_ < 70 Gy without HGI, and 11 patients (42.3%) satisfied it after HGI. The type of catheters used was also analyzed and was not found to have any influence on the rate of variability of the Rectum D_2.0__cm^3^_. On the other hand, factors correlating negatively with the dosimetric effect of HGI were an increased number of interstitial catheters (*P* = 0.003, Fig. 3) as well as L_cranial100%_ (*P* = 0.014) and L_cranial80%_ (*P* = 0.001) (Fig. 4). The association between the rate of variability of the Rectum D_2.0__cm^3^_ and selective clinical factors is summarized in Table 4. The thresholds for number of applicators related to rate of Rectum D_2.0__cm^3^_ variability were not obvious, but in the cases of L_cranial100%_ > 100 mm and L_cranial80%_ > 110 mm, the rate of the Rectum D_2.0__cm^3^_ variability was <30%.

**Table 4.** Analysis of factors influencing Rectum D_2.0__cm^3^_ variability

Parameters	N (patients)		Impact
	Yes	No	*P* value
Prior history of RT			
Number of patients	6	22	*P* = 0.248
R-variability*			
Median (range)	24.5% (0.236–34.5%)	17.0% (3.49–51.7%)	
Use of vaginal cylinder			
Number of patients	6	12	*P* = 0.988
R-variability*			
Median (range)	22.9% (0.236–51.7%)	23.0% (3.95–41.2%)	
Use of tandem and ovoid			
Number of patients	4	24	*P* = 0.289
R-variability*			
Median (range)	29.9% (21.3–41.2%)	21.8% (0.236–51.7%)	

* R-variability: Rate of Rectum D_2.0__cm^3^_ variability = {(pre-HGI Rectum D_2.0__cm^3^_ – post-HGI Rectum D_2.0__cm^3^_)/pre-HGI Rectum D_2.0__cm^3^_} × 100

## DISCUSSION

In RT of gynecological tumors, HDR BT represents an important modality allowing for biological dose escalation while ameliorating conformity through anatomy-oriented 3D treatment planning [7–10]. Notwithstanding this, image-based dose optimization might still pose a clinically relevant challenge when OAR anatomy is hampering the application of sufficient doses, either in the primary or in the recurrence treatment setting [13]. As a result, higher-grade GI or GU toxicity cannot be excluded, even in the case of definitive treatment approaches, with dose escalation offered primarily through 3D HDR BT [37]. In our institution, HGI, which has proven efficacy in reducing late adverse rectal complications in both EBRT and BT for localized prostate cancer [14–23], has been used in gynecologic cancer patients to achieve dose reduction for the rectum. For gynecologic BT, the positive impact of HGI on OAR dosimetry has been documented in a case series of three patients with locally recurrent gynecologic malignancies requiring reirradiation [25]. To the best of our knowledge, this is the largest case series reporting the use of HGI in ISBT/ICBT of gynecologic malignancies, and the results of this analysis indicate that radiation dose exposure of OARs, particularly the rectum, can be safely decreased by HGI. In particular, in primary treatment of cervical cancer, HGI may widen the therapeutic window by increasing the prescription as well as the cumulative biologic dose, analogous to its implementation for RT of organ-confined prostate cancer.

Since tissues may become fibrotic after RT, it seemed reasonable to question whether HGI would be meaningful and feasible in the reirradiation setting. Our series showed that HGI was not only safe to perform in patients who had a prior history of RT (EBRT or BT), but could also reduce the dose to the rectum effectively (Table 4). However, the number of patients who had previous RT was only six, which is relativizing our finding from a statistical point of view without questioning whether HGI could be a supportive tool in the case of repeat RT. In addition, the results of this study indicated that with an increase in the number of interstitial catheters, the dosimetric effect of HGI became less pronounced [Fig. 3]. A conclusive explanation for this phenomenon would be that the high-dose volume overlapping the rectum increased with an increase in the interstitial catheters being implanted perirectally. Since larger tumors may require the implantation of more catheters, it may be concluded that the amount of hyaluronate gel we used in the cases with larger lesions was not sufficient to decisively increase the distance between the rectum and the treatment target. It seems plausible to assume that larger volumes of hyaluronate gel may eliminate this negative correlation by further separating OARs from the planning target volume (PTV). The findings of this study also suggest that the dosimetric impact of HGI was of a lesser magnitude in cases for whom the 100% and 80% prescription isodoses overlapped more rectum volume cranially than caudally in relation to the PTV. A coherent explanation seems to be that the gel catheter needles used initially were not long enough to reach the cranial rectum portions, and therefore a sufficient dose reduction at this level could not be accomplished. As a result, our injection procedure now includes a longer catheter needle (Tuohy needle, Create Medic Company Limited, Yokohama, Japan) of 20 cm length. With this longer application device, it is possible to inject hyaluronate gel to more cranial levels of the perirectal space, thus enhancing the impact of HGI on OAR dosimetry when the PTV is located more cranially within a small pelvis.

There are several limitations in this study. One limitation is that the registration of pre- and post-HGI CT datasets is to some extent associated with intrinsic errors [27]. In our series, the mean error value of registration was 1.84 mm (range: 0.8–2.9 mm), which may be attributable to anatomical changes after HGI. The extent to which this has any clinical relevance cannot be ascertained within the framework of the current work. The other limitation is that our analysis is of a retrospective nature, with inherent limitations and sources of bias. Various modalities of BT and patients with varying backgrounds were included in this report, which makes it difficult to assess the effectiveness of this procedure. Therefore, to determine whether HGI can effectively reduce late rectal toxicity while improving clinical outcomes in gynecologic RT, a prospective study is warranted. Irrespective of the nature of these obstacles, the concept of perirectal gel injection for gynecologic BT is fully plausible, and our initial experience is consistent with recent data showing HGI to be an effective technique for rectal dose reduction in the RT treatment of other intrapelvic tumors [14–23].

In conclusion, the concept of HGI for gynecologic BT is plausible, and our initial experience indicates it to be an effective technique for rectal dose reduction in the RT treatment of intrapelvic tumors. A prospective study is warranted to determine whether HGI can effectively reduce late rectal toxicity while improving clinical outcomes in gynecologic RT.

## ACKNOWLEDGEMENTS

Results from this study were presented at the first meeting of the Federation of Asian Organizations for Radiation Oncology (FARO).

## CONFLICT OF INTEREST

The authors declare they have no competing interests.

## FUNDING

This work was supported by the Japan Agency for Medical Research and Development, AMED, and the National Cancer Center Research and Development Fund (26-A-18 and 26-A-28).

## References

[1] Todo Y, Kato H, Minobe S et al Initial failure site according to primary treatment with or without para-aortic lymphadenectomy in endometrial cancer. Gynecol Oncol 2011;121:314–8. 21315429 10.1016/j.ygyno.2011.01.019

[2] Sartori E, Pasinetti B, Carrara L et al Pattern of failure and value of follow-up procedures in endometrial and cervical cancer patients. Gynecol Oncol 2017;107:241–7. 10.1016/j.ygyno.2007.07.025 17826824

[3] Umezawa R, Murakami N, Nakamura S et al Image-guided interstitial high-dose-rate brachytherapy for locally recurrent uterine cervical cancer: a single-institution study. Brachytherapy 2018;17:368–76. 29275869 10.1016/j.brachy.2017.11.011

[4] Banerjee R, Kamrava M Brachytherapy in the treatment of cervical cancer: a review. Int J Womens Health 2014;6:555–64. 24920937 10.2147/IJWH.S46247 PMC4045176

[5] Viswanathan AN, Lindegaard JC Do intensity-modulated radiation, image-guided radiation, and 3D brachytherapy significantly advance radiotherapeutic management of gynecologic cancers? In: Ledermann JA, Creutzberg CL, Quinn MA (eds). Controversies in the Management of Gynecological Cancers. London: Springer, 2013, 225–35.

[6] Hermesse J, Biver S, Jansen N et al A dosimetric selectivity intercomparison of HDR brachytherapy, IMRT and helical tomotherapy in prostate cancer radiotherapy. Strahlenther Onkol 2009;185:736–42. 19899007 10.1007/s00066-009-2009-5

[7] Shin KH, Kim TH, Cho JK et al CT-guided intracavitary radiotherapy for cervical cancer: comparison of conventional point A plan with clinical target volume-based three-dimensional plan using dose–volume parameters. Int J Radiat Oncol Biol Phys 2006;64:197–204. 16169676 10.1016/j.ijrobp.2005.06.015

[8] Kim H, Beriwal S, Houser C et al Dosimetric analysis of 3D image-guided HDR brachytherapy planning for the treatment of cervical cancer: is point A–based dose prescription still valid in image-guided brachytherapy? Med Dosim 2011;36:166–70. 20488690 10.1016/j.meddos.2010.02.009

[9] Murakami N, Kato T, Miyamoto Y et al Salvage high-dose-rate interstitial brachytherapy for pelvic recurrent cervical carcinoma after hysterectomy. Anticancer Res 2016;36:2413–21. 27127151

[10] De Ieso PB, Mullassery V, Shrimali R et al Image-guided vulvovaginal interstitial brachytherapy in the treatment of primary and recurrent gynecological malignancies. Brachytherapy 2012;11:306–10. 21996537 10.1016/j.brachy.2011.08.002

[11] Yamazaki H, Inoue T, Ikeda H et al High-dose-rate remote afterloading intestinal radiotherapy employing the template technique for recurrent cancer in the pelvic area. Strahlenther Onkol 1993;169:481–5. 8356507

[12] Mazeron R, Gilmore J, Champoudry J et al Volumetric evaluation of an alternative bladder point in brachytherapy for locally advanced cervical cancer. Strahlenther Onkol 2014;190:41–7. 24240504 10.1007/s00066-013-0463-6

[13] Dörr W, Herrmann T, Baumann M Application of organ tolerance dose-constraints in clinical studies in radiation oncology. Strahlenther Onkol 2014;190:621–7. 24604558 10.1007/s00066-014-0613-5

[14] Kishi K, Sato M, Sonomura T et al Reirradiation of prostate cancer with rectum preservation: eradicative high-dose-rate brachytherapy with natural type hyaluronate injection. Brachytherapy 2012;11:144–8. 21820975 10.1016/j.brachy.2011.06.006

[15] Guimas V, Quivrin M, Bertaut A et al Focal or whole-gland salvage prostate brachytherapy with iodine seeds with or without a rectal spacer for postradiotherapy local failure: how best to spare the rectum? Brachytherapy 2016;15:406–11. 27317949 10.1016/j.brachy.2016.03.014

[16] Prada PJ, Gonzalez H, Menéndez C et al Transperineal injection of hyaluronic acid in the anterior perirectal fat to decrease rectal toxicity from radiation delivered with low-dose-rate brachytherapy for prostate cancer patients. Brachytherapy 2009;8:210–7. 19213607 10.1016/j.brachy.2008.11.010

[17] Mariados N, Sylvester J, Shah D et al Hydrogel spacer prospective multicenter randomized controlled pivotal trial: dosimetric and clinical effects of perirectal spacer application in men undergoing prostate image guided intensity modulated radiation therapy. Int J Radiat Oncol Biol Phys 2015;92:971–7. 26054865 10.1016/j.ijrobp.2015.04.030

[18] Song DY, Herfarth KK, Uhl M et al A multi-institutional clinical trial of rectal dose reduction via injected polyethylene-glycol hydrogel during intensity modulated radiation therapy for prostate cancer: analysis of dosimetric outcomes. Int J Radiat Oncol Biol Phys 2013;87:81–7. 23414766 10.1016/j.ijrobp.2012.12.019 PMC3737267

[19] Eckert F, Alloussi S, Paulsen F et al Prospective evaluation of a hydrogel spacer for rectal separation in dose-escalated intensity-modulated radiotherapy for clinically localized prostate cancer. BMC Cancer 2013;13:27–34. 23336502 10.1186/1471-2407-13-27 PMC3558402

[20] Mahal BA, Ziehr DR, Hyatt AS et al Use of a rectal spacer with low-dose-rate brachytherapy for treatment of prostate cancer in previously irradiated patients: initial experience and short-term results. Brachytherapy 2014;13:442–9. 24880584 10.1016/j.brachy.2014.05.001

[21] Nguyen PL, Devlin PM, Beard CJ et al High-dose-rate brachytherapy for prostate cancer in a previously radiated patient with polyethylene glycol hydrogel spacing to reduce rectal dose: case report and review of the literature. Brachytherapy 2013;12:77–83. 22543116 10.1016/j.brachy.2012.03.005

[22] Prada PJ, Jimenez I, Gonzalez-Suarez H et al High-dose-rate interstitial brachytherapy as monotherapy in one fraction and transperineal hyaluronic acid injection into the perirectal fat for the treatment of favorable stage prostate cancer: treatment description and preliminary results. Brachytherapy 2012;11:105–10. 21917528 10.1016/j.brachy.2011.05.003

[23] Alongi F, Cozzi L, Arcangeli S et al Linac based SBRT for prostate cancer in 5 fractions with VMAT and flattening filter free beams: preliminary report of a phase II study. Radiat Oncol 2013;8:171. 23835141 10.1186/1748-717X-8-171 PMC3718706

[24] Kishi K, Mabuchi Y, Sonomura T et al Eradicative brachytherapy with hyaluronate gel injection into pararectal space in treatment of bulky vaginal stump recurrence of uterine cancer. J Radiat Res 2012;53:601–7. 22843626 10.1093/jrr/rrs015 PMC3393341

[25] Viswanathan AN, Damato AL, Nguyen PL et al Novel use of a hydrogel spacer permits reirradiation in otherwise incurable recurrent gynecologic cancers. J Clin Oncol 2013;31:446–7. 10.1200/JCO.2012.47.9931 PMC5795666 24145342

[26] Damato AL, Kassick M, Viswanathan AN et al Rectum and bladder spacing in cervical cancer brachytherapy using a novel injectable hydrogel compound. Brachytherapy 2017;16:949–55. 28619385 10.1016/j.brachy.2017.04.236

[27] Zukauskaite R, Brink C, Hansen CR et al Open source deformable image registration system for treatment planning and recurrence CT scans: validation in the head and neck region. Strahlenther Onkol 2016;192:545–51. 27323754 10.1007/s00066-016-0998-4

[28] Mazeron R, Fokdal LU, Kirchheiner K et al Dose–volume effect relationships for late rectal morbidity in patients treated with chemoradiation and MRI-guided adaptive brachytherapy for locally advanced cervical cancer: results from the prospective multicenter EMBRACE study. Radiother Oncol 2016;120:412–9. 27396811 10.1016/j.radonc.2016.06.006

[29] Kato S, Tran DN, Ohno T et al CT-based 3D dose–volume parameter of the rectum and late rectal complication in patients with cervical cancer treated with high-dose-rate intracavitary brachytherapy. J Radiat Res 2010;51:215–21. 20339256 10.1269/jrr.09118

[30] Assenholt MS, Vestergarrd A, Kallehauge JF et al Proof of principle: applicator-guided stereotactic IMRT boost in combination with 3D MRI-based brachytherapy in locally advanced cervical cancer. Brachytherapy 2014;13:361–8. 24656732 10.1016/j.brachy.2014.02.003

[31] Murakami N, Kasamatsu T, Sumi M et al Vaginal tolerance of CT based image-guided high-dose rate interstitial brachytherapy for gynecological malignancies. Radiat Oncol 2014;9:31–9. 24456669 10.1186/1748-717X-9-31 PMC3909309

[32] Murakami N, Kobayashi K, Kato T et al The role of interstitial brachytherapy in the management of primary radiation therapy for uterine cervical cancer. J Contemp Brachytherapy 2016;5:391–8. 10.5114/jcb.2016.62938 PMC5116446 27895680

[33] Itami J, Hara R, Kozuka T et al Transperineal high-dose-rate interstitial brachytherapy in the management of gynecologic malignancies. Strahlenther Onkol 2003;179:737–41. 14605742 10.1007/s00066-003-1069-1

[34] Benjamini Y, Braun H John W. Tukey’s contributions to multiple comparisons. Ann Stat 2002;30:1576–94.

[35] Student The probable error of a mean. Biometrika 1908;6:1–25.

[36] Ebina Y, Yaegashi N, Katabuchi H et al Japan Society of Gynecologic Oncology guidelines 2011 for the treatment of uterine cervical cancer. Int J Clin Oncol 2015;20:240–8. 25800808 10.1007/s10147-015-0806-7

[37] Georg P, Boni A, Ghabuous A et al Time course of late rectal- and urinary bladder side effects after MRI-guided adaptive brachytherapy for cervical cancer. Strahlenther Onkol 2013;189:535–40. 23703404 10.1007/s00066-013-0365-7

